# Supplementary material for: Modeling mitochondrial inheritance enables high-precision single-cell lineage tracing in humans
Source: bioRxiv. 2026 Feb 18:2026.02.12.705660. Preprint. [Version 2] doi: 10.64898/2026.02.12.705660 (PMC12918977; doi:10.64898/2026.02.12.705660)
Supplement: Supplement 1 [file NIHPP2026.02.12.705660v2-supplement-1.pdf]

Figure S1

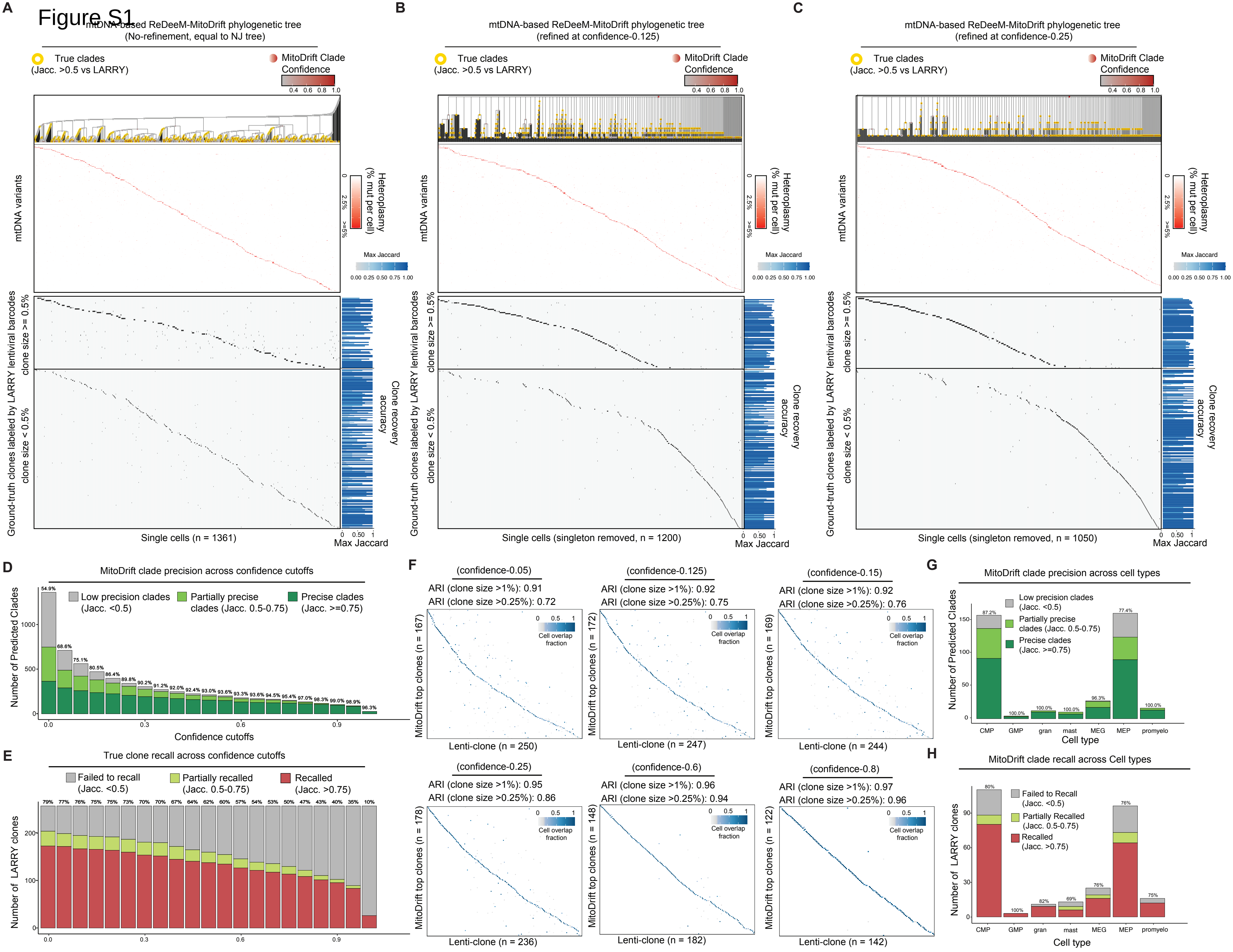
A
mtDNA-based ReDeeM-MitoDrift phylogenetic tree
(No-refinement, equal to NJ tree)
True clades
(Jacc. >0.5 vs LARRY)
MitoDrift Clade
Confidence
mtDNA variants
Heteroplasmy
(% mut per cell)
Max Jaccard
Ground-truth clones labeled by LARRY lentiviral barcodes
clone size >= 0.5%
clone size < 0.5%
Clone recovery
accuracy
Single cells (n = 1361)
Max Jaccard
B
mtDNA-based ReDeeM-MitoDrift phylogenetic tree
(refined at confidence-0.125)
Single cells (singleton removed, n = 1200)
C
mtDNA-based ReDeeM-MitoDrift phylogenetic tree
(refined at confidence-0.25)
Single cells (singleton removed, n = 1050)
D
MitoDrift clade precision across confidence cutoffs
Low precision clades
(Jacc. <0.5)
Partially precise
clades (Jacc. 0.5-0.75)
Precise clades
(Jacc. >=0.75)
Number of Predicted Clades
Confidence cutoffs
E
True clone recall across confidence cutoffs
Failed to recall
(Jacc. <0.5)
Partially recalled
(Jacc. 0.5-0.75)
Recalled
(Jacc. >0.75)
Number of LARRY clones
F
(confidence-0.05)
ARI (clone size >1%): 0.91
ARI (clone size >0.25%): 0.72
MitoDrift top clones (n = 167)
Lenti-clone (n = 250)
(confidence-0.125)
ARI (clone size >1%): 0.92
ARI (clone size >0.25%): 0.75
MitoDrift top clones (n = 172)
Lenti-clone (n = 247)
(confidence-0.15)
ARI (clone size >1%): 0.92
ARI (clone size >0.25%): 0.76
MitoDrift top clones (n = 169)
Lenti-clone (n = 244)
(confidence-0.25)
ARI (clone size >1%): 0.95
ARI (clone size >0.25%): 0.86
MitoDrift top clones (n = 178)
Lenti-clone (n = 236)
(confidence-0.6)
ARI (clone size >1%): 0.96
ARI (clone size >0.25%): 0.94
MitoDrift top clones (n = 148)
Lenti-clone (n = 182)
(confidence-0.8)
ARI (clone size >1%): 0.97
ARI (clone size >0.25%): 0.96
MitoDrift top clones (n = 122)
Lenti-clone (n = 142)
Cell overlap
fraction
G
MitoDrift clade precision across cell types
Low precision clades
(Jacc. <0.5)
Partially precise
clades (Jacc. 0.5-0.75)
Precise clades
(Jacc. >=0.75)
Number of Predicted Clades
CMP
GMP
gran
mast
MEG
MEP
promyelo
Cell type
H
MitoDrift clade recall across Cell types
Failed to Recall
(Jacc. <0.5)
Partially Recalled
(Jacc. 0.5-0.75)
Recalled
(Jacc. >0.75)
Number of LARRY clones
Cell type

bioRxiv preprint doi: https://doi.org/10.64898/2026.02.12.705660; this version posted February 18, 2026. The copyright holder for this preprint (which was not certified by peer review) is the author/funder, who has granted bioRxiv a license to display the preprint in perpetuity. It is made available under aCC-BY 4.0 International license.

Figure S1. Clade precision and clone recall in the LARRY benchmark.

(A–C) mtDNA-based ReDeeM-MitoDrift phylogenetic trees for the LARRY benchmark shown without confidence refinement (A) or refined at confidence thresholds $\tau = 0.125$ (B) and $\tau = 0.25$ (C). Trees are annotated with true clades based on overlap with LARRY barcode clones (Jaccard ≥ 0.5; yellow) and posterior clade confidence (color bar); heatmaps show mitochondrial variants across cells (top) and LARRY barcode-defined ground-truth clone identities (bottom), with per-barcode clonal recovery accuracy summarized as the maximum Jaccard overlap (right). (D) Precision of inferred clades with different confidence cutoffs, stratified by clade–clone overlap (false positives: Jaccard < 0.5; true positives: Jaccard 0.5–0.75 and ≥0.75). (E) Number of LARRY barcode clones recovered at each cutoff, stratified by best-matching inferred clade overlap (not recovered: Jaccard < 0.5; recovered: Jaccard ≥0.5 and ≥0.75). (F) Concordance between MitoDrift root-clone assignments and LARRY clone identities across confidence cutoffs, visualized as clone–clone overlap matrices (color indicates cell overlap fraction) and summarized by adjusted Rand index (ARI). (G) Clade precision stratified by annotated cell type. (H) Clone recall stratified by annotated cell type.

bioRxiv preprint doi: https://doi.org/10.64898/2026.02.12.705660; this version posted February 18, 2026. The copyright holder for this preprint (which was not certified by peer review) is the author/funder, who has granted bioRxiv a license to display the preprint in perpetuity. It is made available under aCC-BY 4.0 International license.

Figure S2

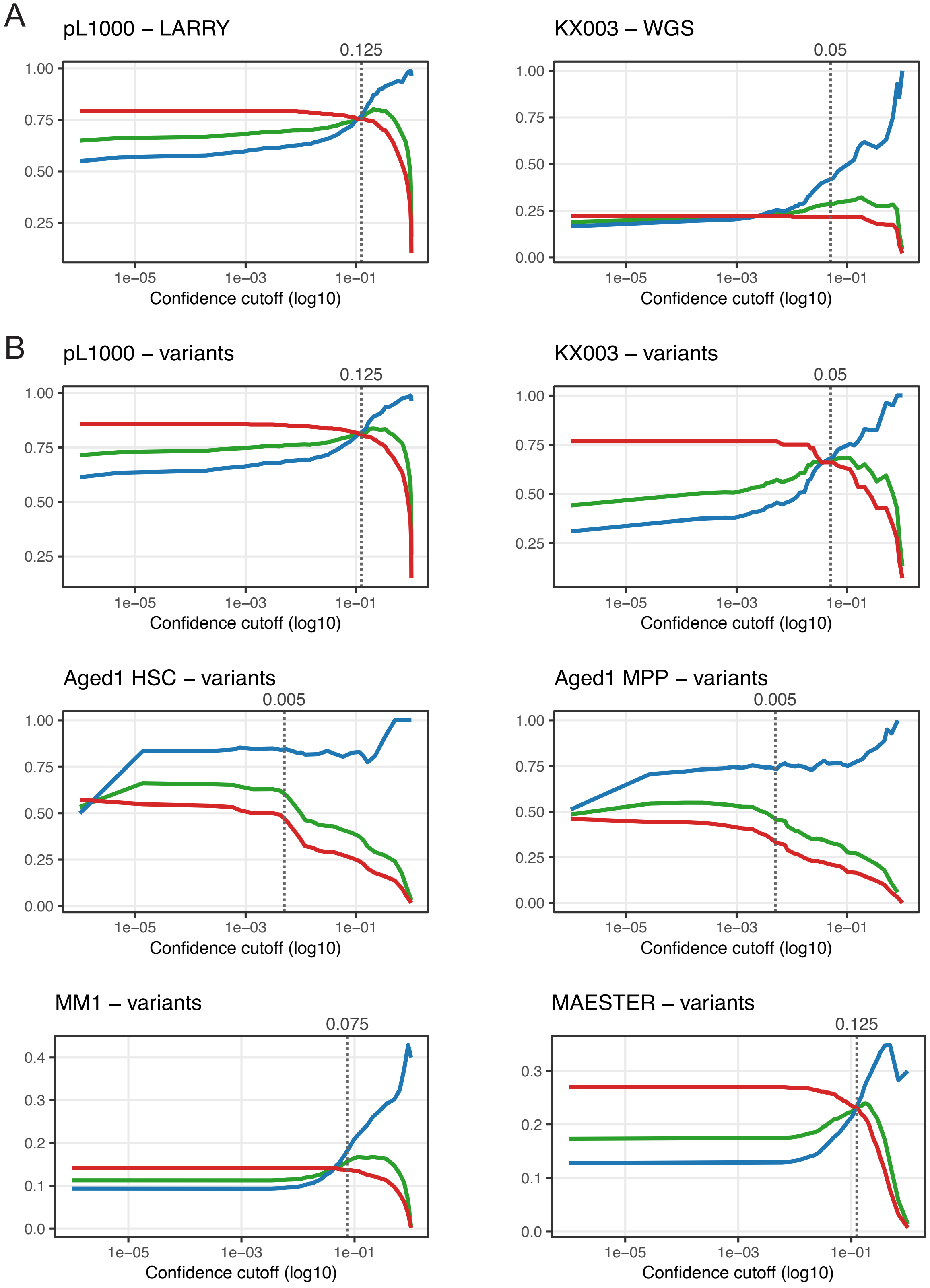

bioRxiv preprint doi: https://doi.org/10.64898/2026.02.12.705660; this version posted February 18, 2026. The copyright holder for this preprint (which was not certified by peer review) is the author/funder, who has granted bioRxiv a license to display the preprint in perpetuity. It is made available under aCC-BY 4.0 International license.

Figure S2. Confidence-cutoff diagnostic curves across datasets.

(A) Precision, recall, and F1 score as a function of the posterior clade-support cutoff (τ; x-axis, log10 scale) when evaluating confidence-refined MitoDrift tree against external ground truth in LARRY (pL1000; barcode clones) and the WGS benchmark (KX003; nuclear SNV phylogeny clades). (B) For datasets without external ground truth, an internal diagnostic curve based on mtDNA variant consistency: variant carrier sets are treated as “true” clones, and precision/recall/F1 are computed from the overlap (Jaccard) between variant carrier sets and predicted clades. Panels show example curves for the LARRY and WGS datasets (for comparison to A), as well as for Aged 1 HSC/MPP, MM1, and the MAESTER-CHIP dataset. In all panels, the dashed vertical line indicates the selected cutoff used for downstream analyses (value annotated above each panel).

bioRxiv preprint doi: https://doi.org/10.64898/2026.02.12.705660; this version posted February 18, 2026. The copyright holder for this preprint (which was not certified by peer review) is the author/funder, who has granted bioRxiv a license to display the preprint in perpetuity. It is made available under aCC-BY 4.0 International license.

Figure S3

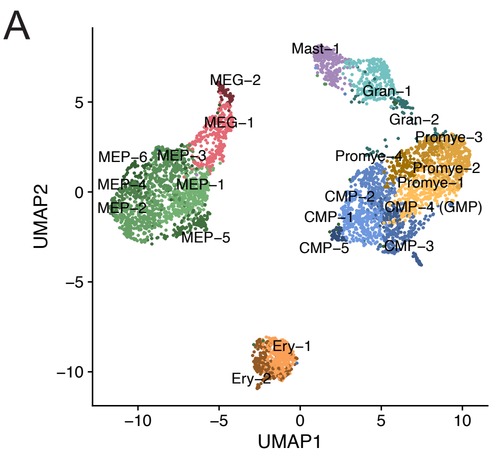

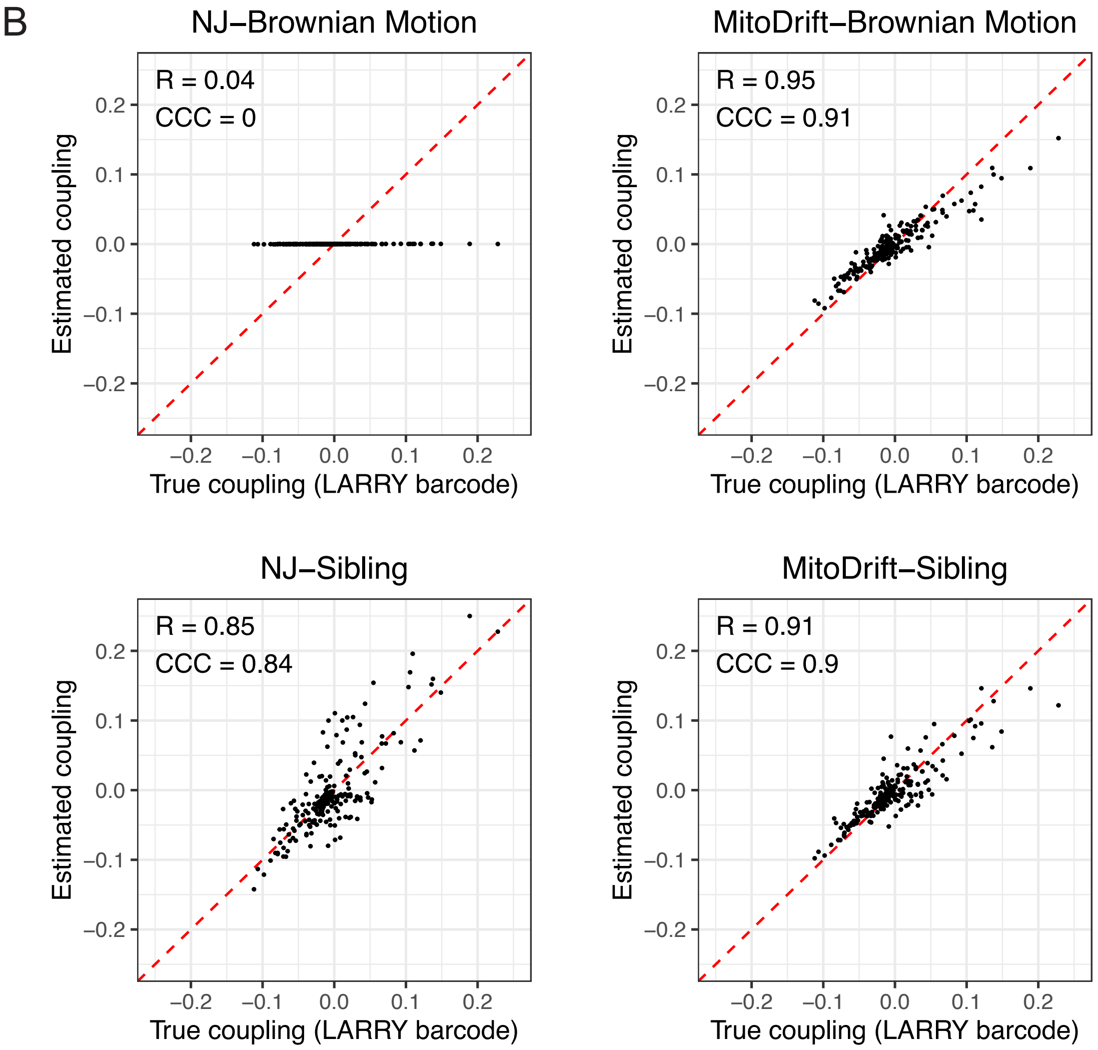

bioRxiv preprint doi: https://doi.org/10.64898/2026.02.12.705660; this version posted February 18, 2026. The copyright holder for this preprint (which was not certified by peer review) is the author/funder, who has granted bioRxiv a license to display the preprint in perpetuity. It is made available under aCC-BY 4.0 International license.

Figure S3. Cell-state map and barcode ground truth validation of coupling estimates.

(A) UMAP of the LARRY benchmark with annotated hematopoietic cell states used for lineage-coupling analysis. (B) Agreement between estimated lineage coupling and barcode-defined coupling ("true coupling") using Neighbor-Joining (NJ) and refined MitoDrift trees, tested in combination with two different phylogenetic distance models: Brownian Motion and Sibling. Dashed lines indicate identity; Pearson's correlation (R) and Lin's Concordance Correlation Coefficient (CCC) are shown for each comparison.

bioRxiv preprint doi: https://doi.org/10.64898/2026.02.12.705660; this version posted February 18, 2026. The copyright holder for this preprint (which was not certified by peer review) is the author/funder, who has granted bioRxiv a license to display the preprint in perpetuity. It is made available under aCC-BY 4.0 International license.

Figure S4

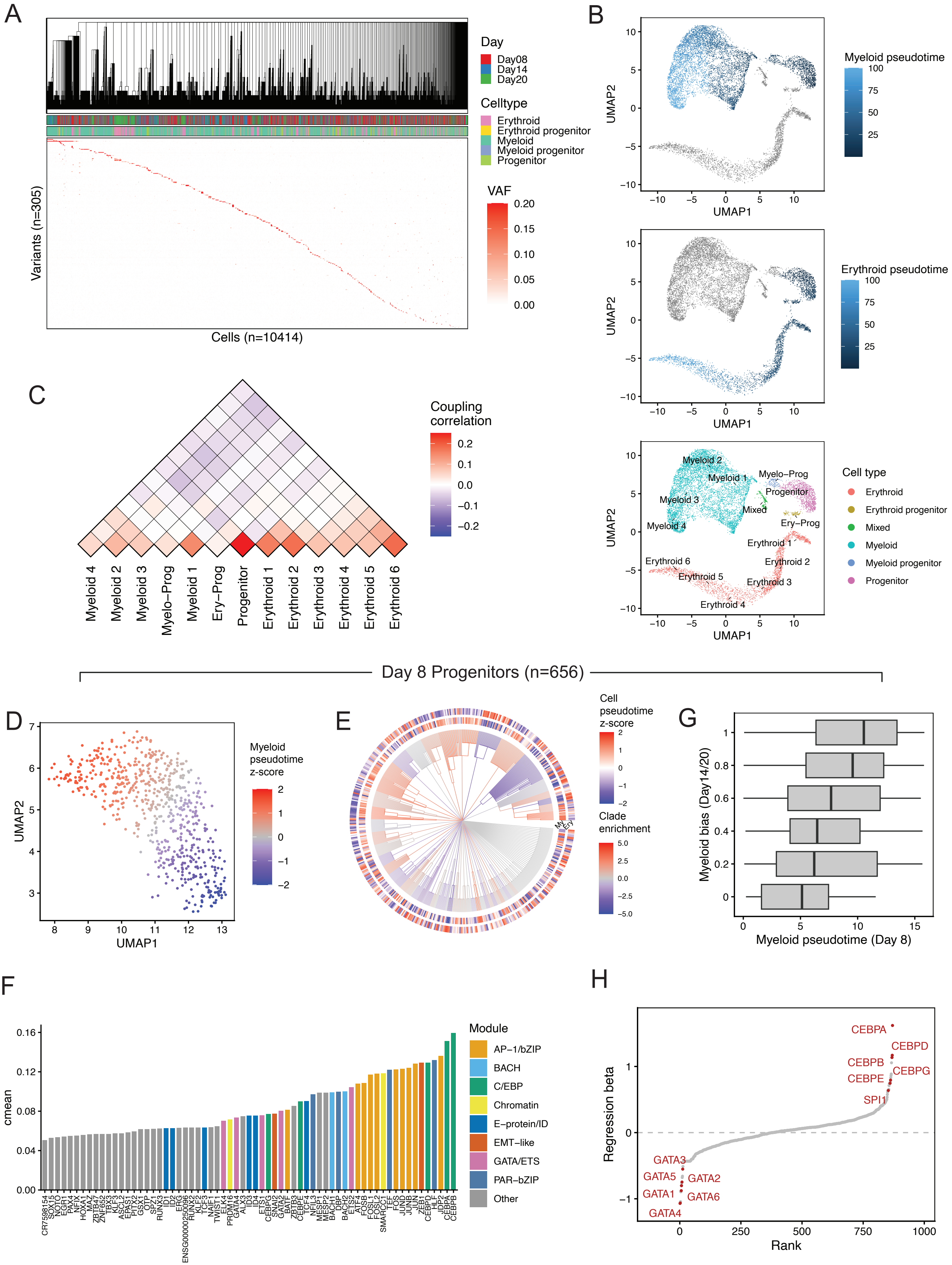

bioRxiv preprint doi: https://doi.org/10.64898/2026.02.12.705660; this version posted February 18, 2026. The copyright holder for this preprint (which was not certified by peer review) is the author/funder, who has granted bioRxiv a license to display the preprint in perpetuity. It is made available under aCC-BY 4.0 International license.

Figure S4. Reanalysis of in vitro CD34+ differentiation mtscATAC-seq data.

(A) Cell lineage tree (refined at confidence threshold $\tau = 0.125$) inferred from the Lareau et al. mtscATAC-seq in vitro CD34+ differentiation dataset, juxtaposed with variant allele frequencies (VAF; color scale) across cells (n = 10,414) and mtDNA variants (n = 930); VAF heatmap shows variants detected in ≥15 cells. Cells are ordered by the inferred lineage, with top annotations indicating collection day and annotated cell type. (B) UMAP embedding of the mtscATAC-seq dataset colored by inferred myeloid pseudotime, erythroid pseudotime, and annotated cell types and states. (C) Lineage coupling matrix summarizing pairwise coupling correlations between annotated cell types and states inferred by PATH based on the MitoDrift-inferred lineage tree. (D) UMAP of day 8 progenitors colored by myeloid pseudotime (rescaled z-scores within day 8). (E) MitoDrift lineage tree for day 8 progenitors (refined at confidence threshold $\tau = 0.125$; singletons omitted), with cells annotated by myeloid and erythroid pseudotime (outer ring) and clade-level z-scores for myeloid pseudotime. (F) Phylogenetic signal (cmean) of chromVAR TF and motif activity features within day 8 progenitors, grouped by functional module. (G) Association between day 8 myeloid pseudotime and a myeloid-bias score defined by the fraction of myeloid descendants among day 14 and day 20 cells; boxplots show the distribution of day 8 myeloid pseudotime across bins of the myeloid-bias score. (H) Ranked regression coefficients for TF and motif activity features in day 8 progenitors associated with myeloid-bias at days 14 and 20, with representative regulators highlighted.

bioRxiv preprint doi: https://doi.org/10.64898/2026.02.12.705660; this version posted February 18, 2026. The copyright holder for this preprint (which was not certified by peer review) is the author/funder, who has granted bioRxiv a license to display the preprint in perpetuity. It is made available under aCC-BY 4.0 International license.

Figure S5

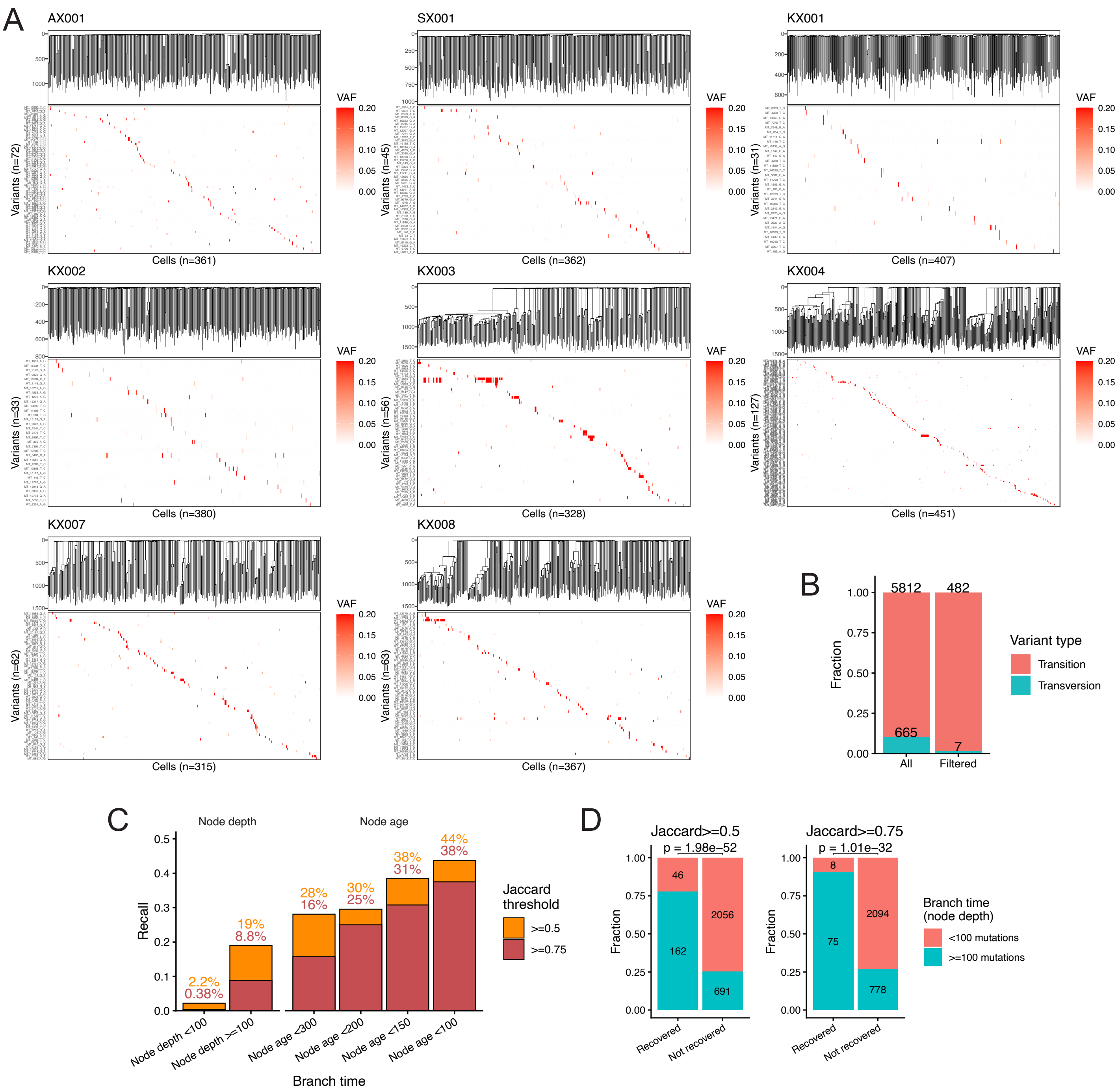

bioRxiv preprint doi: https://doi.org/10.64898/2026.02.12.705660; this version posted February 18, 2026. The copyright holder for this preprint (which was not certified by peer review) is the author/funder, who has granted bioRxiv a license to display the preprint in perpetuity. It is made available under aCC-BY 4.0 International license.

Figure S5. WGS-derived cell lineage trees and mtDNA variants in the in vivo benchmark samples. (A) WGS-derived lineage trees (top) and mtDNA variant allele frequency (VAF) heatmaps (bottom) across single cells for each benchmark sample, with cells ordered by the WGS-derived lineage and mtDNA variants shown on the y-axis (VAF color scale). (B) Fractions of transition and transversion substitutions across all called mtDNA variants and after filtering to variants retained for phylogeny inference. (C) Ground-truth clade recall by MitoDrift (without confidence refinement) as a function of branch time (node depth), evaluated at Jaccard overlap thresholds (≥0.5 and ≥0.75). (D) Fractions of recovered versus not recovered internal nodes at each Jaccard threshold, stratified by branch-time category (<100 versus ≥100 mutations); Fisher's exact test *P* values are shown above each comparison.

bioRxiv preprint doi: https://doi.org/10.64898/2026.02.12.705660; this version posted February 18, 2026. The copyright holder for this preprint (which was not certified by peer review) is the author/funder, who has granted bioRxiv a license to display the preprint in perpetuity. It is made available under aCC-BY 4.0 International license.

Figure S6

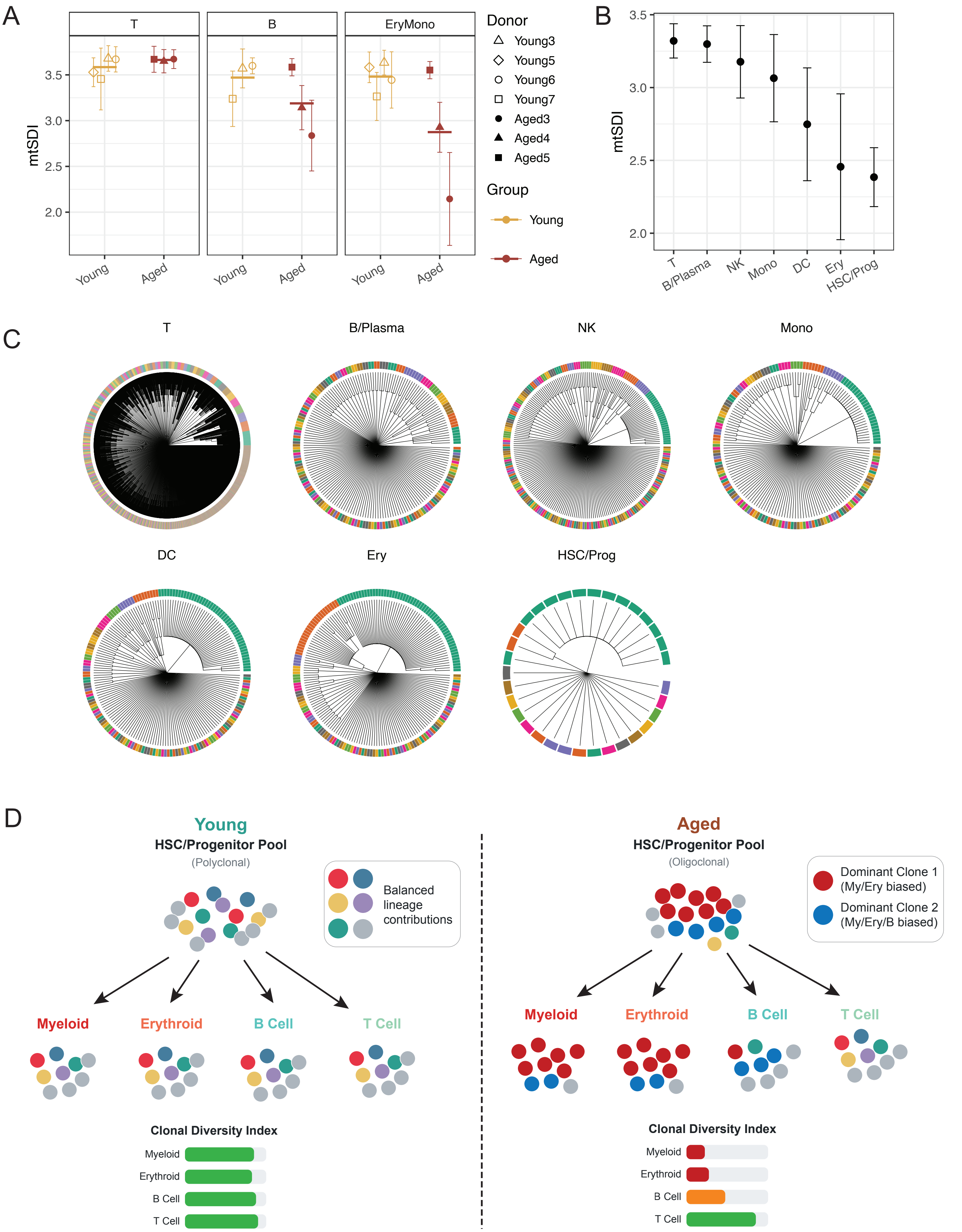

bioRxiv preprint doi: https://doi.org/10.64898/2026.02.12.705660; this version posted February 18, 2026. The copyright holder for this preprint (which was not certified by peer review) is the author/funder, who has granted bioRxiv a license to display the preprint in perpetuity. It is made available under aCC-BY 4.0 International license.

Figure S6. Cell-type-resolved clonal diversity in additional datasets.

(A) Shannon diversity index (mtSDI) comparing young versus aged donors across hematopoietic compartments in the donor-multiplexed ReDeeM cohort; computed from 10 random subsamples of 45 cells per cell type per donor (mean ± 95% CI). (B) Mean mtSDI across major hematopoietic compartments in the CHIP donor (Miller et al.) profiled using MAESTER, with error bars representing bootstrap-based 95% confidence intervals. (C) Confidence-refined MitoDrift phylogenies (refined at confidence threshold $\tau = 0.125$) for each hematopoietic cell type in the MAESTER dataset, illustrating cell-type-specific differences in clonal structure. (D) Conceptual schematic contrasting polyclonal versus oligoclonal HSC/progenitor pools and how lineage-biased contributions from dominant clones can differentially reduce clonal diversity across mature lineages.

bioRxiv preprint doi: https://doi.org/10.64898/2026.02.12.705660; this version posted February 18, 2026. The copyright holder for this preprint (which was not certified by peer review) is the author/funder, who has granted bioRxiv a license to display the preprint in perpetuity. It is made available under aCC-BY 4.0 International license.

Figure S7

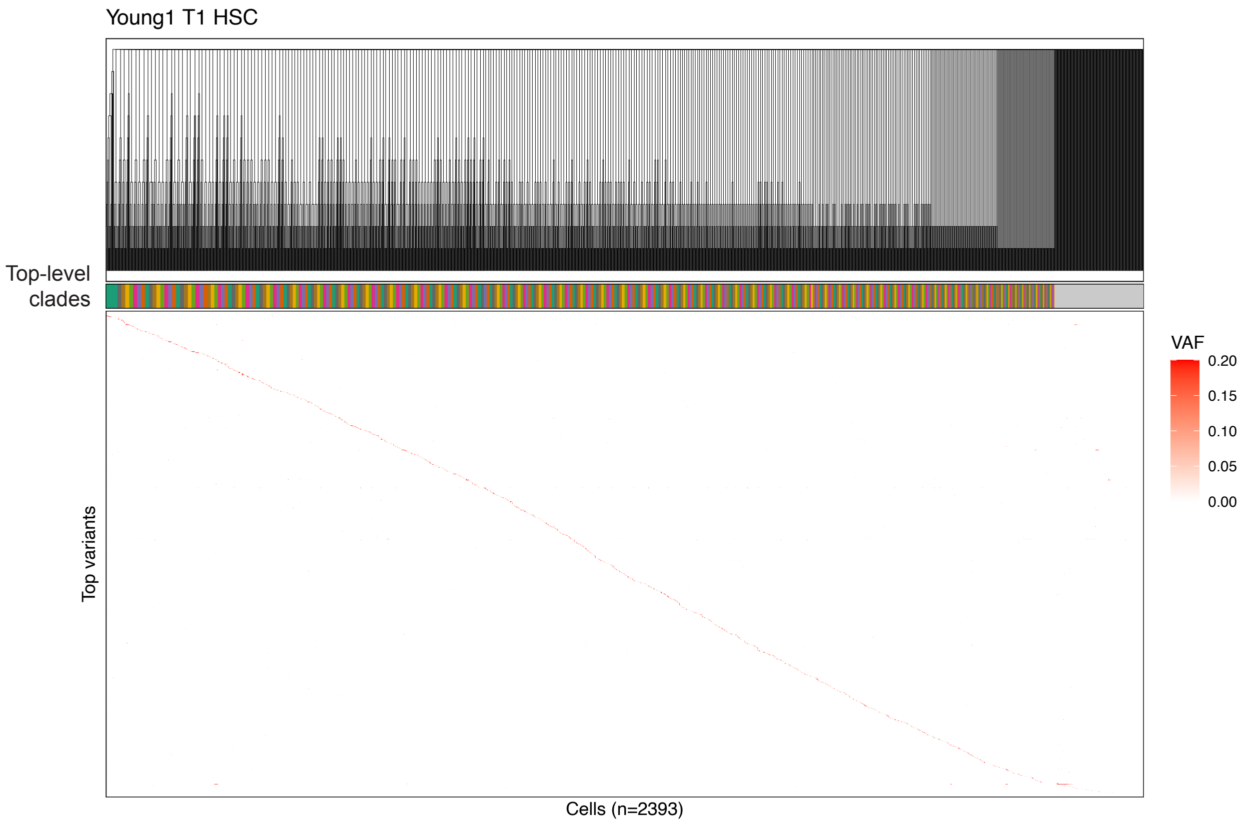

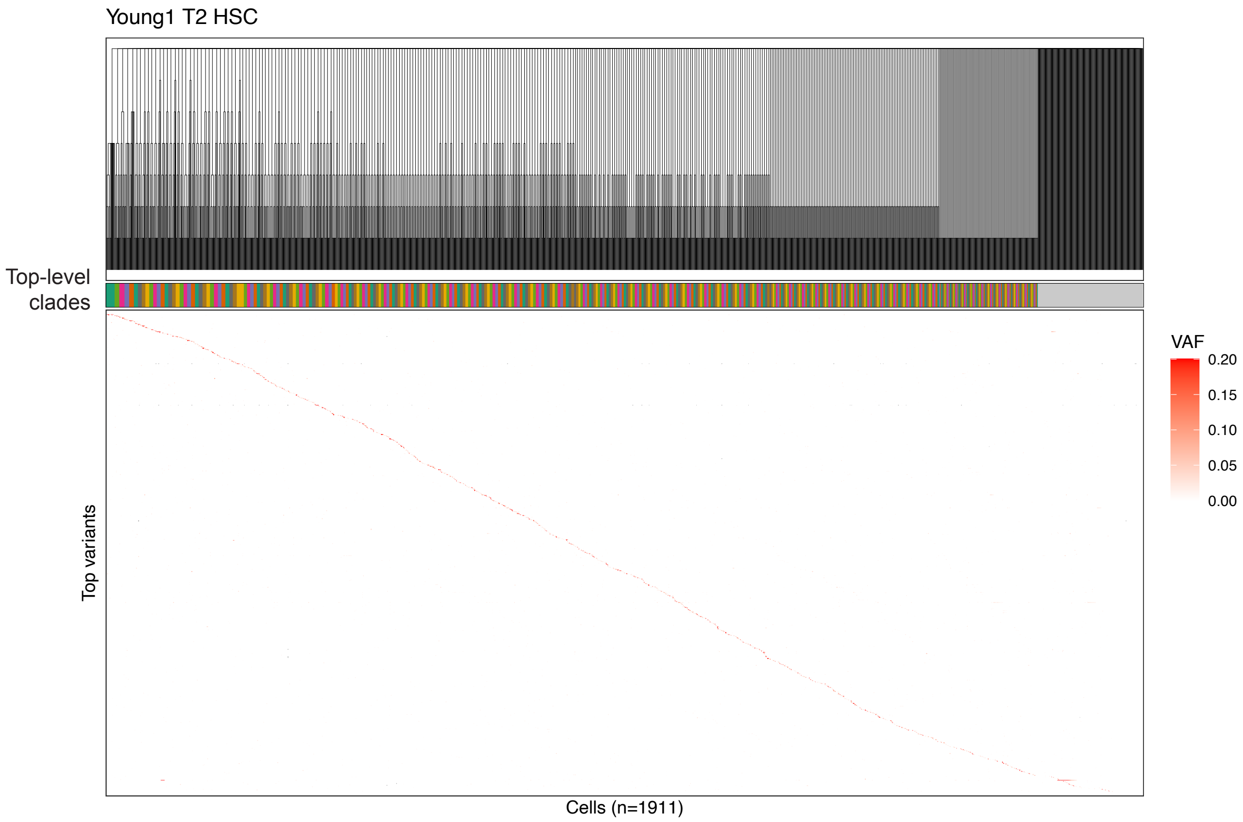

bioRxiv preprint doi: https://doi.org/10.64898/2026.02.12.705660; this version posted February 18, 2026. The copyright holder for this preprint (which was not certified by peer review) is the author/funder, who has granted bioRxiv a license to display the preprint in perpetuity. It is made available under aCC-BY 4.0 International license.

Figure S7. Inferred phylogenies and mtDNA variant patterns in Young1 HSC samples.

Heatmaps of mitochondrial variant allele frequency (VAF) across single HSCs from donor Young1 at timepoint T1 (top) and timepoint T2 (bottom). Cells are ordered by the lineage tree inferred by MitoDrift (top dendrogram). The colored annotation bar indicates inferred clade assignments (refined using the expected-misassignment criterion with $\epsilon = 0.002$; Methods). Heatmap color denotes per-cell VAF for each mtDNA variant.

bioRxiv preprint doi: https://doi.org/10.64898/2026.02.12.705660; this version posted February 18, 2026. The copyright holder for this preprint (which was not certified by peer review) is the author/funder, who has granted bioRxiv a license to display the preprint in perpetuity. It is made available under aCC-BY 4.0 International license.

Figure S8

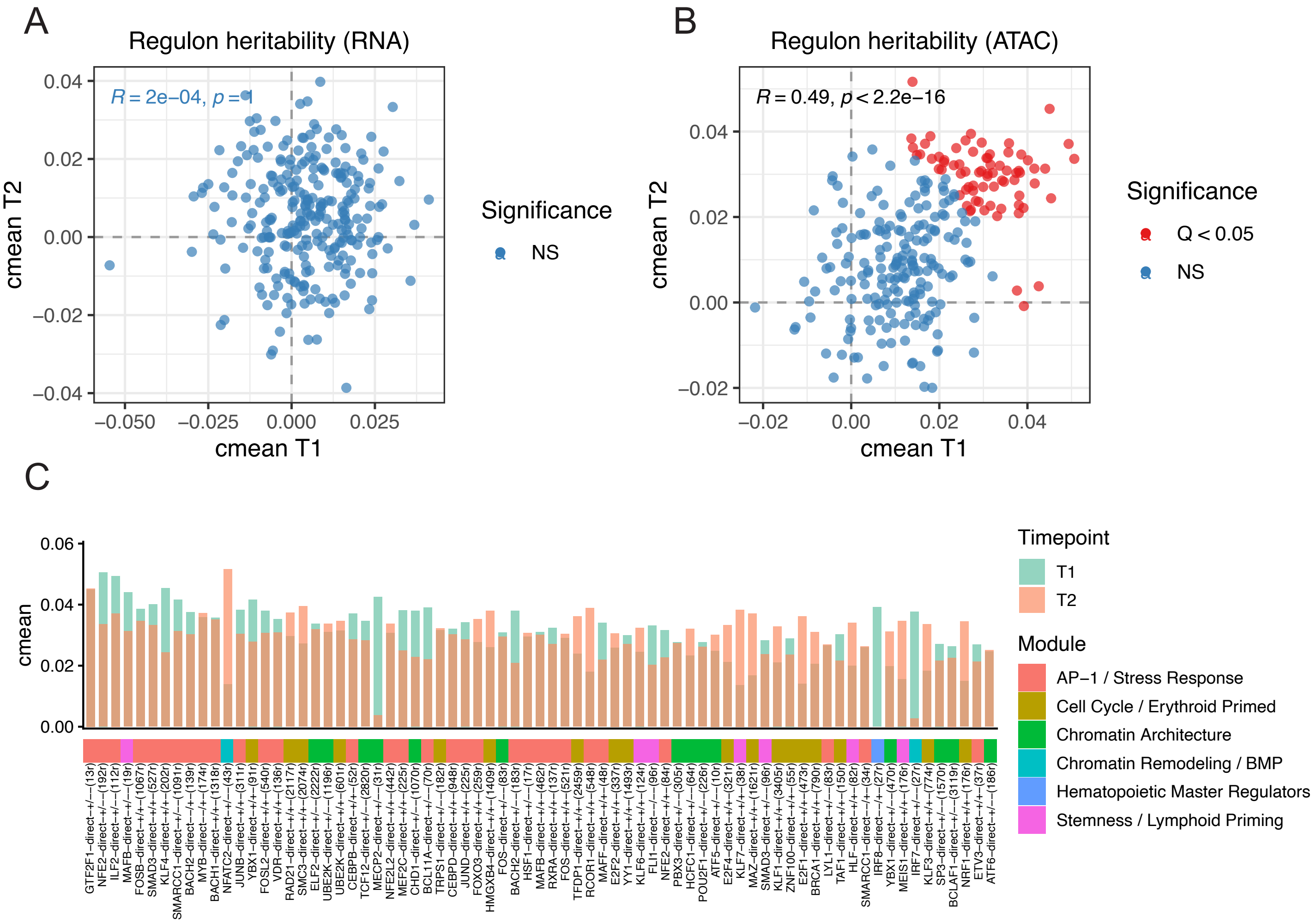

bioRxiv preprint doi: https://doi.org/10.64898/2026.02.12.705660; this version posted February 18, 2026. The copyright holder for this preprint (which was not certified by peer review) is the author/funder, who has granted bioRxiv a license to display the preprint in perpetuity. It is made available under aCC-BY 4.0 International license.

Figure S8. Regulon heritability across timepoints.

(A) Scatter plot comparing regulon heritability estimates (cmean) between two longitudinal timepoints (T1 versus T2) using RNA-derived regulon activity scores. (B) Same analysis using ATAC-derived regulon activity scores. Each point denotes a regulon; points are colored by statistical significance in the combined analysis ($Q < 0.05$). (C) Per-regulon heritability (cmean) at each timepoint for regulons significant in the combined analysis (ATAC-based activity scores), with module assignments indicated.

bioRxiv preprint doi: https://doi.org/10.64898/2026.02.12.705660; this version posted February 18, 2026. The copyright holder for this preprint (which was not certified by peer review) is the author/funder, who has granted bioRxiv a license to display the preprint in perpetuity. It is made available under aCC-BY 4.0 International license.

Figure S9

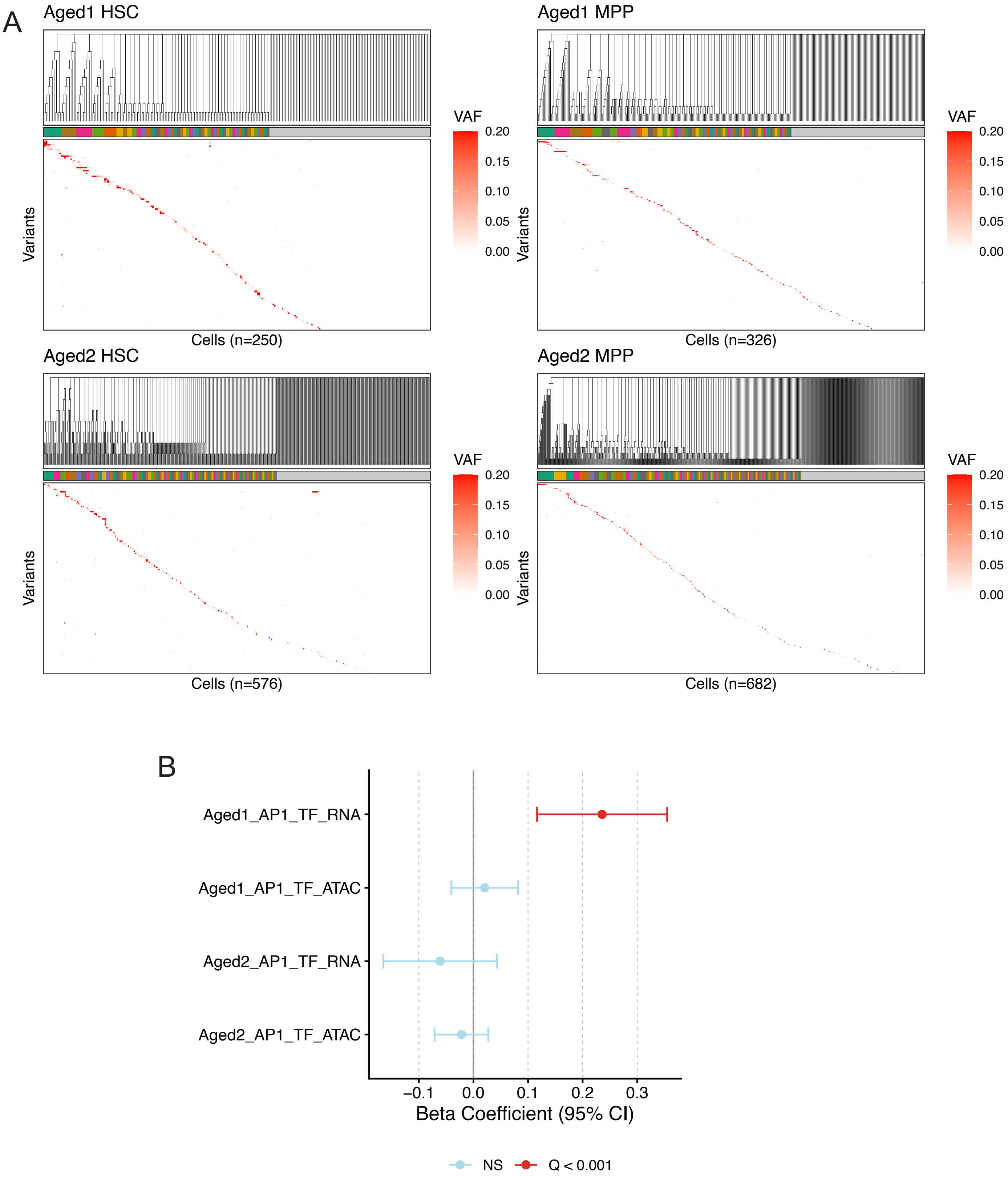

bioRxiv preprint doi: https://doi.org/10.64898/2026.02.12.705660; this version posted February 18, 2026. The copyright holder for this preprint (which was not certified by peer review) is the author/funder, who has granted bioRxiv a license to display the preprint in perpetuity. It is made available under aCC-BY 4.0 International license.

Figure S9. Aged donor HSC/MPP lineage structure and association of AP-1 activity with clonal size.

(A) MitoDrift phylogenies (refined at confidence threshold $\tau = 0.005$ for Aged 1, $\tau = 0.01$ for Aged 2) and mtDNA VAF heatmaps for aged donor HSCs and MPPs, with cells ordered by the inferred lineage. Clone assignments (colored bar) are shown above each heatmap. (B) Regression coefficients (beta ± 95% CI) relating average AP-1 TF activity to inferred clone size across aged donors, shown separately for RNA- and ATAC-derived TF activity for each donor.

bioRxiv preprint doi: https://doi.org/10.64898/2026.02.12.705660; this version posted February 18, 2026. The copyright holder for this preprint (which was not certified by peer review) is the author/funder, who has granted bioRxiv a license to display the preprint in perpetuity. It is made available under aCC-BY 4.0 International license.

Figure S10

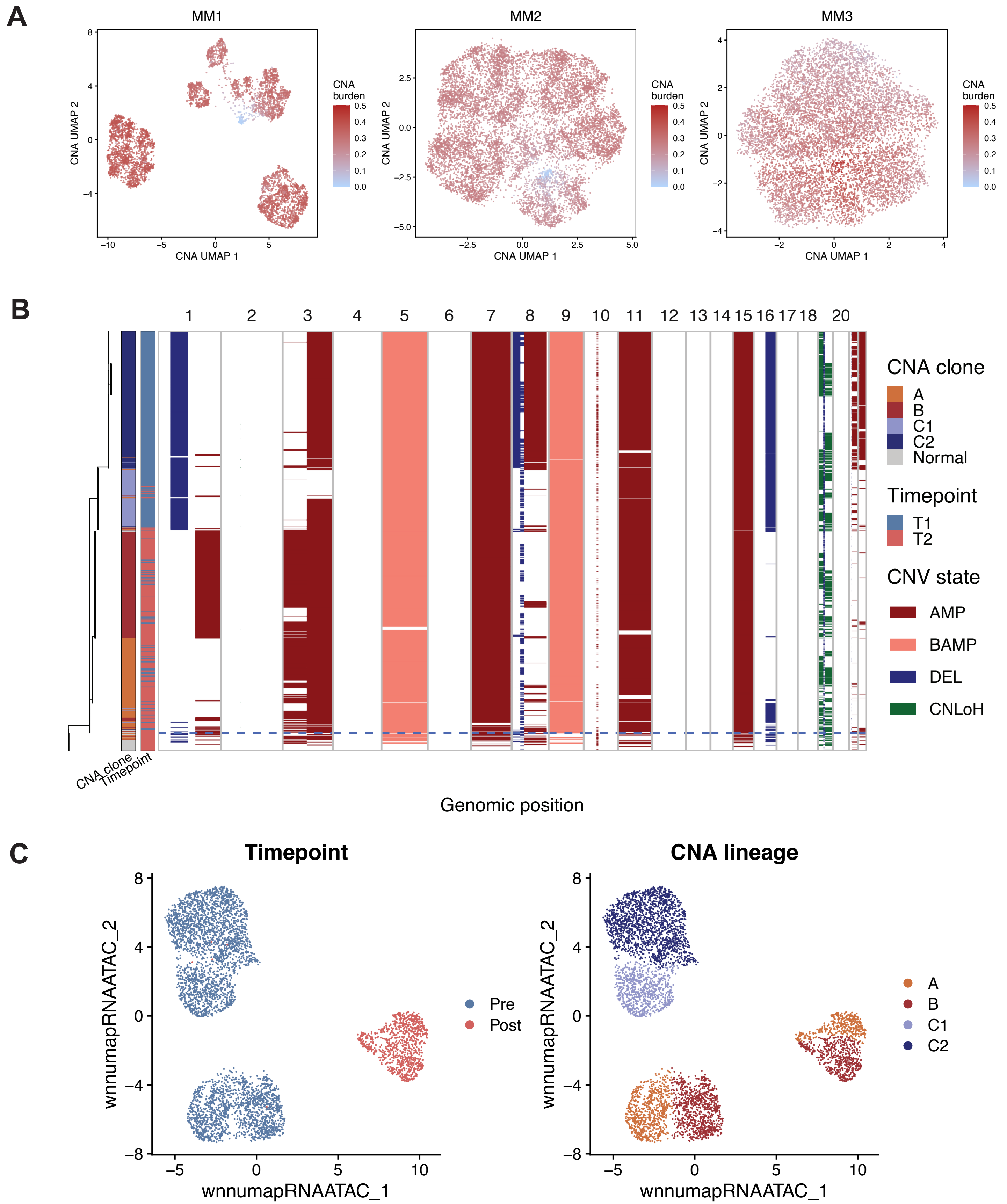

bioRxiv preprint doi: https://doi.org/10.64898/2026.02.12.705660; this version posted February 18, 2026. The copyright holder for this preprint (which was not certified by peer review) is the author/funder, who has granted bioRxiv a license to display the preprint in perpetuity. It is made available under aCC-BY 4.0 International license.

Figure S10. Copy number analysis in myeloma multiome data.

(A) CNA-based UMAP projections for plasma cells colored by Numbat-inferred CNA burden for each donor. (B) Heatmap of Numbat-inferred copy-number states for MM1 across the genome for single cells; CNA states are annotated as amplifications (AMP), balanced amplifications (BAMP), deletions (DEL), and copy-neutral loss of heterozygosity (CNLOH). (C) wnnUMAP projection of MM1 malignant plasma cells colored by sampling timepoint and by inferred CNA lineage.

bioRxiv preprint doi: https://doi.org/10.64898/2026.02.12.705660; this version posted February 18, 2026. The copyright holder for this preprint (which was not certified by peer review) is the author/funder, who has granted bioRxiv a license to display the preprint in perpetuity. It is made available under aCC-BY 4.0 International license.

Figure S11

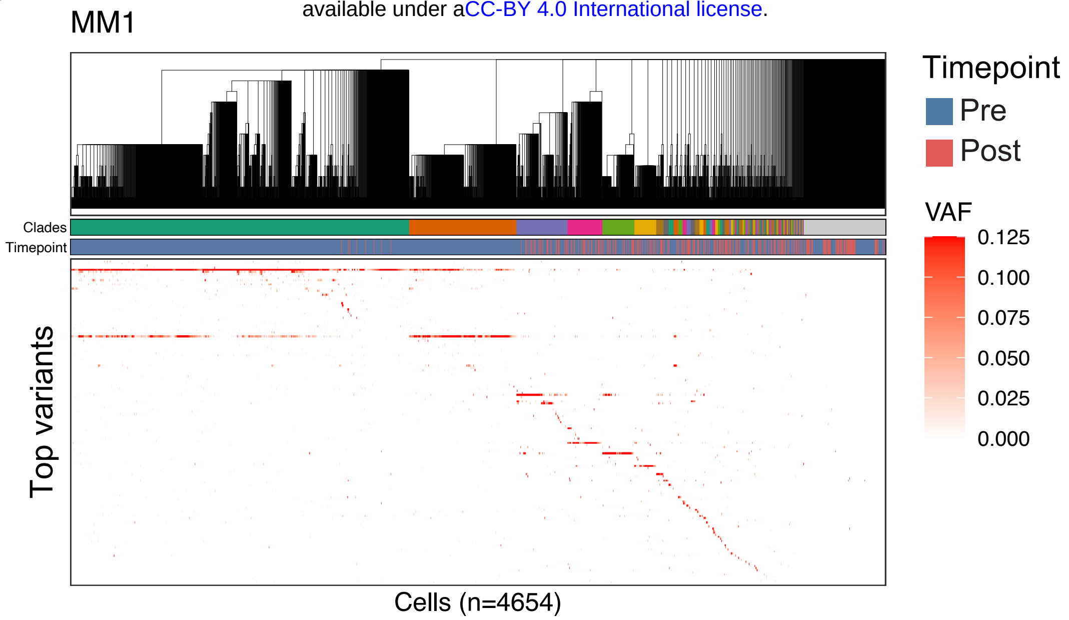

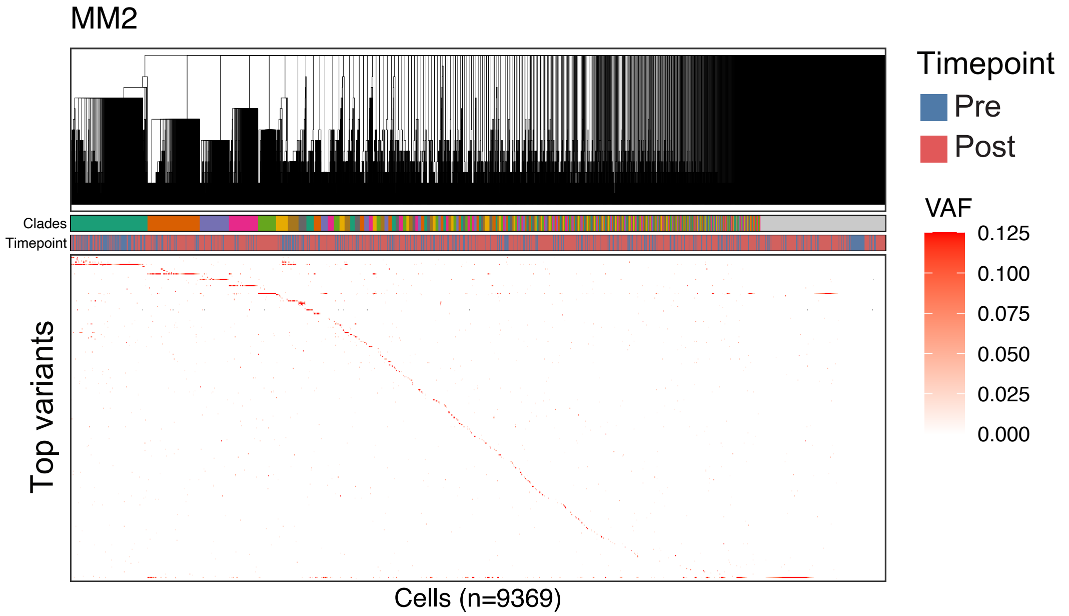

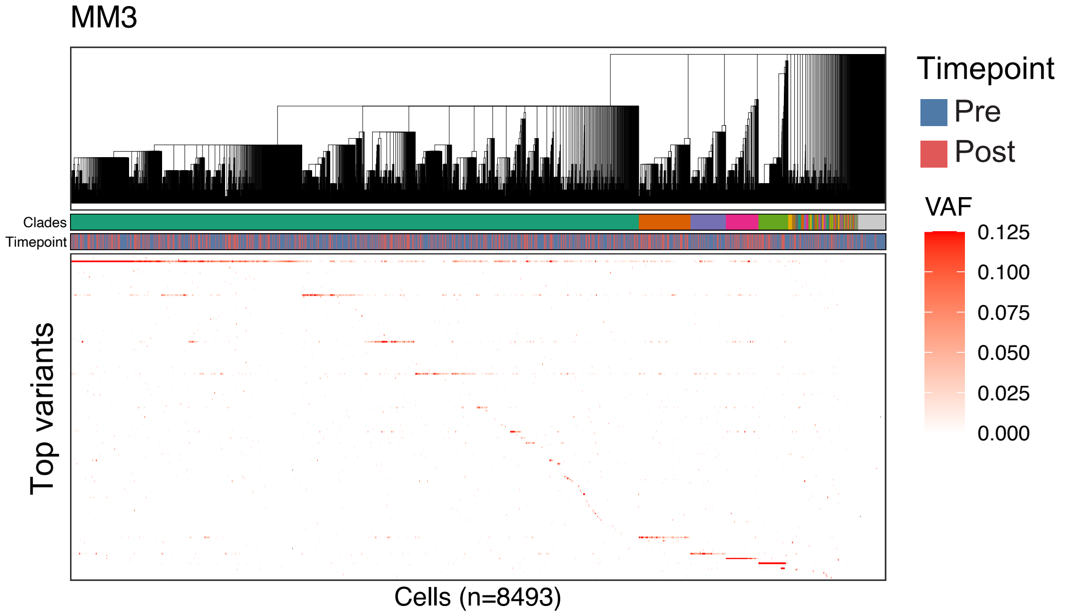

bioRxiv preprint doi: https://doi.org/10.64898/2026.02.12.705660; this version posted February 18, 2026. The copyright holder for this preprint (which was not certified by peer review) is the author/funder, who has granted bioRxiv a license to display the preprint in perpetuity. It is made available under aCC-BY 4.0 International license.

Figure S11. Inferred phylogenies and mtDNA variant patterns in multiple myeloma samples.

Heatmaps of mitochondrial variant allele frequency (VAF) across samples from three patients (MM1–3; top to bottom). Cells are ordered by the lineage tree inferred by MitoDrift (top dendrogram; refined at confidence threshold $\tau = 0.075$). The colored annotation bar indicates MitoDrift clone assignments. Heatmap color denotes per-cell VAF for each mtDNA variant.

bioRxiv preprint doi: https://doi.org/10.64898/2026.02.12.705660; this version posted February 18, 2026. The copyright holder for this preprint (which was not certified by peer review) is the author/funder, who has granted bioRxiv a license to display the preprint in perpetuity. It is made available under aCC-BY 4.0 International license.

Figure S12

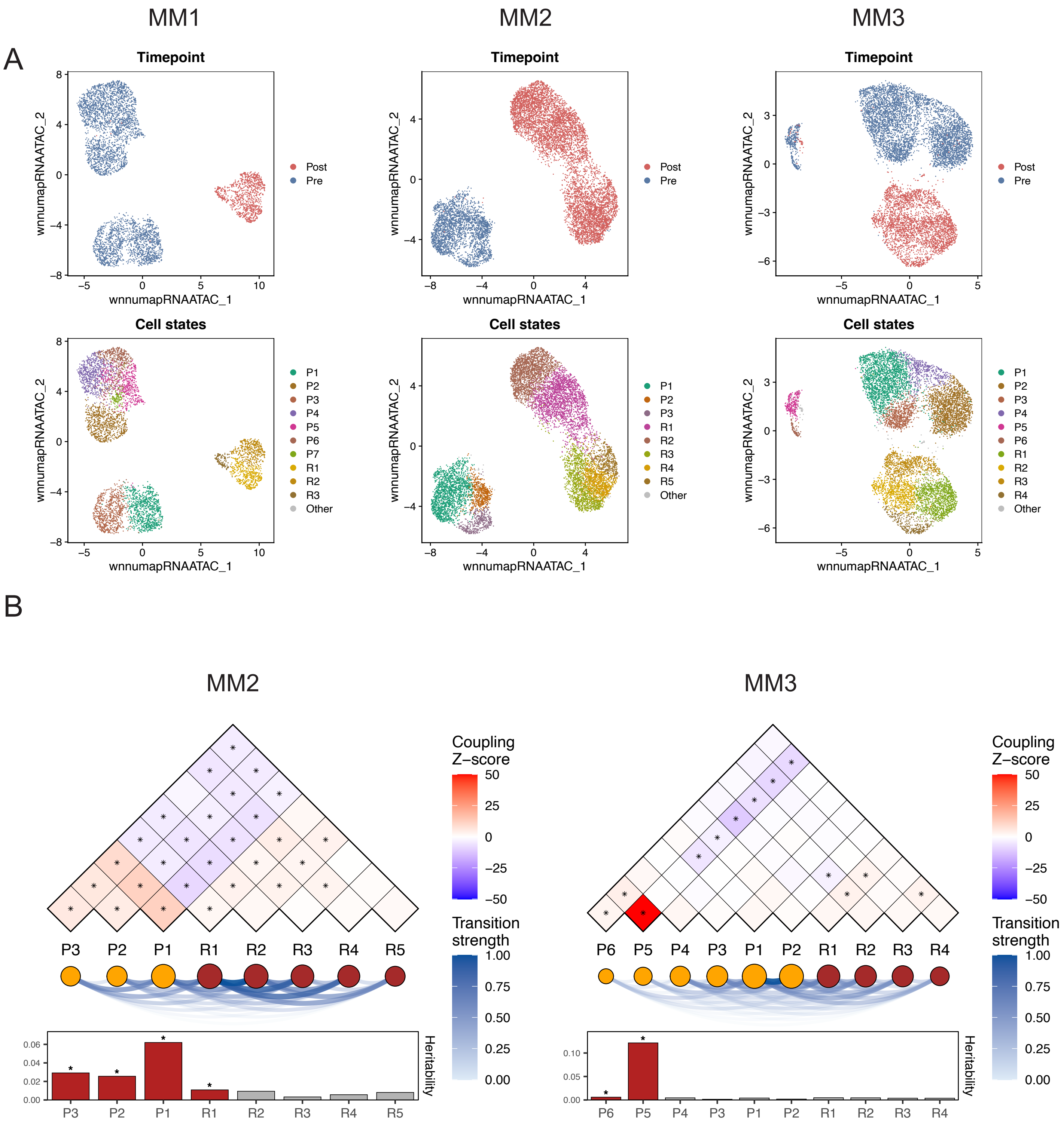

bioRxiv preprint doi: https://doi.org/10.64898/2026.02.12.705660; this version posted February 18, 2026. The copyright holder for this preprint (which was not certified by peer review) is the author/funder, who has granted bioRxiv a license to display the preprint in perpetuity. It is made available under aCC-BY 4.0 International license.

Figure S12. Lineage–state coupling analyses across additional multiple myeloma patients.

(A) wnnUMAP projections of malignant plasma cells from three multiple myeloma patients (MM1–MM3) colored by sampling timepoint (pre- versus post-treatment; top) and by annotated malignant cell states (bottom). (B) Cell state coupling summary for MM2 and MM3, showing lineage coupling between malignant cell states (pairwise coupling Z-scores), inferred transition strengths between states, and per-state heritability on the cell lineage tree.

bioRxiv preprint doi: https://doi.org/10.64898/2026.02.12.705660; this version posted February 18, 2026. The copyright holder for this preprint (which was not certified by peer review) is the author/funder, who has granted bioRxiv a license to display the preprint in perpetuity. It is made available under aCC-BY 4.0 International license.

Figure S13

A

mut-filter strategies	signal_density	%_cells_with_mut
redeem−filter2	1746	73
redeem−no−1umi	938	55
mgatk−baseline	810	50
mgatk−het>0.07	572	39

mut-filter strategies	signal_density	%_cells_with_mut
redeem−filter2	1746	73
redeem−filter1	3681	84

mut-filter strategies	signal_density	%_cells_with_mut
redeem−filter2	1746	73
redeem−het>0.02	1087	60
redeem−het>0.04	803	50
redeem−het>0.06	636	42
redeem−het>0.08	542	38
redeem−het>0.10	472	34

B

C

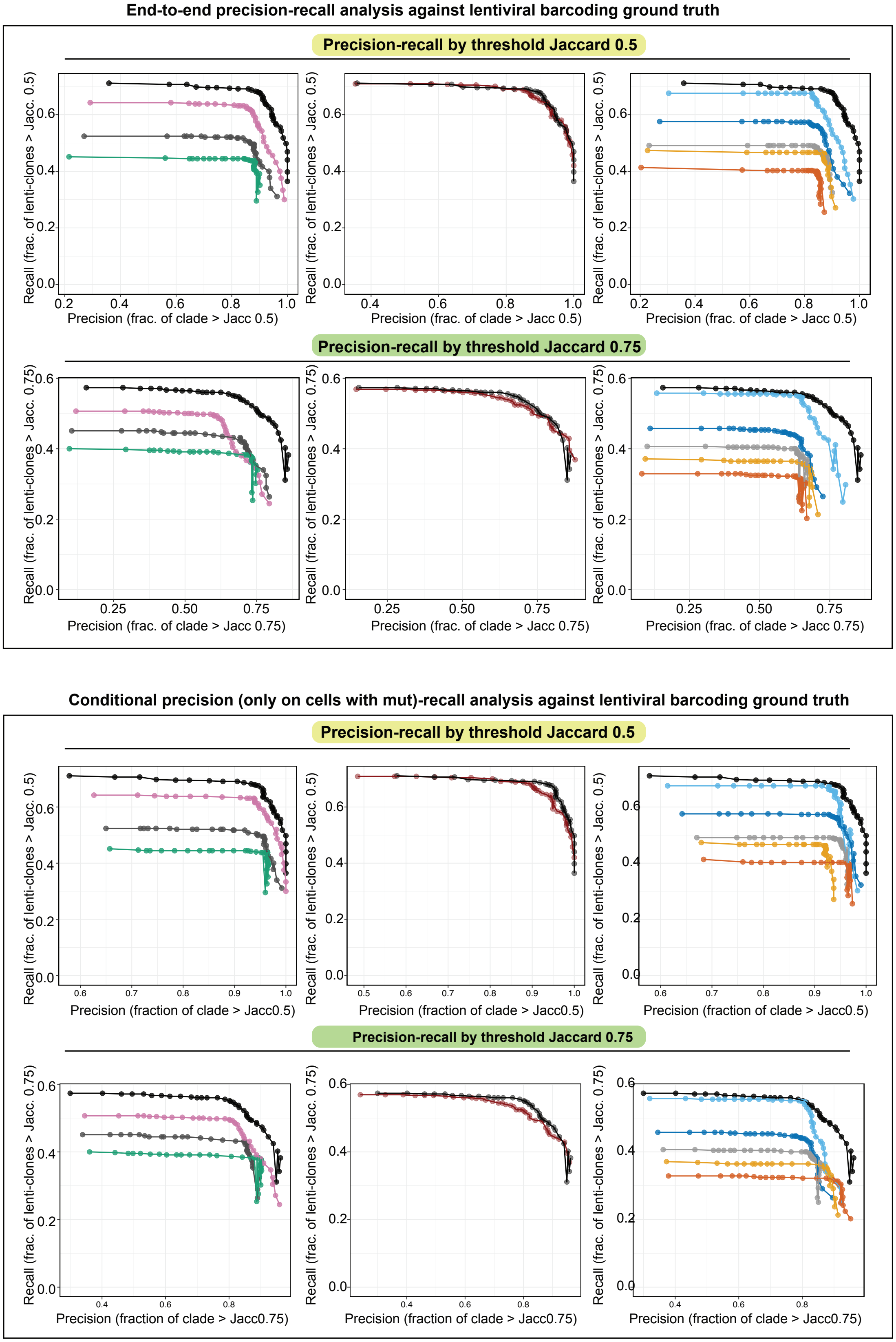

bioRxiv preprint doi: https://doi.org/10.64898/2026.02.12.705660; this version posted February 18, 2026. The copyright holder for this preprint (which was not certified by peer review) is the author/funder, who has granted bioRxiv a license to display the preprint in perpetuity. It is made available under aCC-BY 4.0 International license.

Figure S13. Benchmarking the impact of mtDNA variant filtering on lineage reconstruction performance.

(A) Summary of mutation retention after applying each predefined variant-filtering strategy. Signal_density reports the number of retained mutation observations per 1,000 cells after filtering, where a mutation observation is a retained cell–variant entry. %_cells_with_mut reports the percentage of cells retaining ≥1 mutation after filtering (i.e., evaluable cells). (B) End-to-end precision–recall benchmarking of phylogenetic reconstruction across filtering strategies, evaluated against lentiviral barcoding (LARRY) ground-truth clone labels (clone size ≥2) at Jaccard overlap thresholds of 0.5 and 0.75. The three comparison groups (left to right) are (i) ReDeeM vs mgatk preprocessing, where ReDeeM is evaluated under ReDeeM filter-2 and ReDeeM filter-2 with 1UMI-supported mutations removed, and mgatk is evaluated under its baseline settings and an additional heteroplasmy ≥0.07 filtering regime recommended in prior work (Lareau et al., 2021, Nat. Biotech.); (ii) ReDeeM filter-1 vs filter-2; and (iii) ReDeeM filter-2 under increasing heteroplasmy thresholds. Precision and recall are computed on the same full set of cells for each condition ("end-to-end"), thus capturing both clade accuracy and signal dropout effects. (C) Conditional-precision analysis of precision–recall using the same benchmarking framework as in (B), but computing precision only among evaluable cells (cells retaining ≥1 mutation observation after filtering) while retaining full-cell recall. This isolates clade accuracy among retained-signal cells from mutation dropout effects. Across panels (B–C), inferred clades are obtained by collapsing the MitoDrift phylogeny across a sweep of confidence refinement thresholds (τ). Precision is defined as the fraction of inferred clades whose best-matching ground-truth clone exceeds the indicated Jaccard threshold, and recall is defined as the fraction of ground-truth clones whose best-matching inferred clade exceeds the threshold. Curves are averaged across 10 matched clone-based subsets reused across all conditions to control for subset composition effects, with matched inference parameters across conditions.

bioRxiv preprint doi: https://doi.org/10.64898/2026.02.12.705660; this version posted February 18, 2026. The copyright holder for this preprint (which was not certified by peer review) is the author/funder, who has granted bioRxiv a license to display the preprint in perpetuity. It is made available under aCC-BY 4.0 International license.

Figure S14

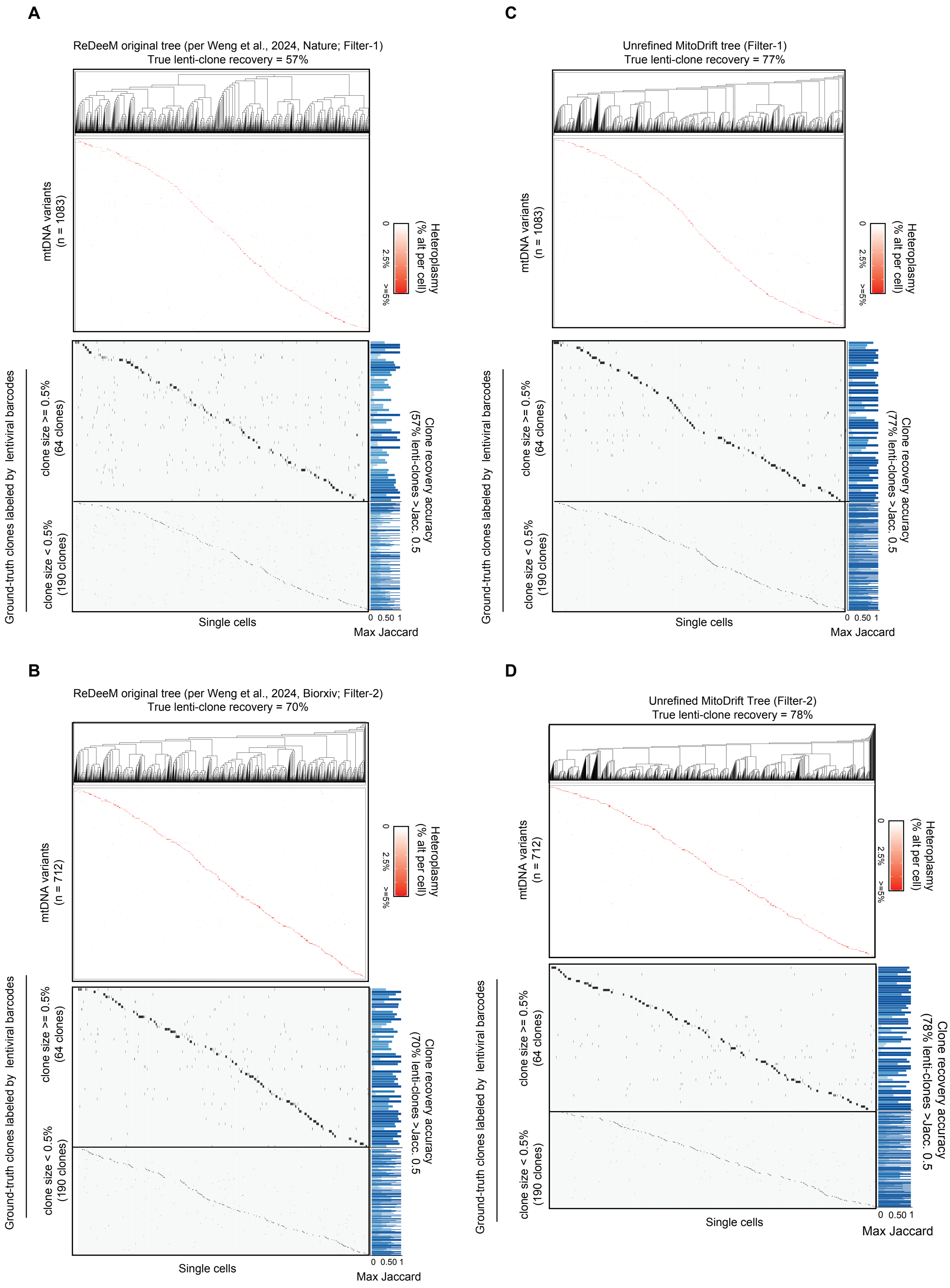

bioRxiv preprint doi: https://doi.org/10.64898/2026.02.12.705660; this version posted February 18, 2026. The copyright holder for this preprint (which was not certified by peer review) is the author/funder, who has granted bioRxiv a license to display the preprint in perpetuity. It is made available under aCC-BY 4.0 International license.

Figure S14. Ground-truth clone recovery in original ReDeeM and unrefined MitoDrift trees.

(A–B) Original ReDeeM tree (Neighbor-Joining on binarized variants). Phylogeny constructed using the ReDeeM workflow: mtDNA variants binarized (presence/absence), pairwise weighted Jaccard distances computed, and Neighbor-Joining (NJ) tree inferred. (A) Filter-1 (Weng et al., 2024, Nature). (B) Filter-2 (stricter filtering; Weng et al., 2024, bioRxiv). (C–D) Unrefined MitoDrift tree (NJ backbone, pre-calibration). Phylogeny represents the NJ backbone used in MitoDrift before posterior-based clade collapsing. The workflow is: mtDNA variants are not binarized; Manhattan distances are computed from continuous heteroplasmy profiles, followed by NJ inference. (C) Filter-1. (D) Filter-2. Each panel displays: top, inferred NJ phylogeny; middle, mtDNA variant heteroplasmy heatmap (rows, variants; columns, cells ordered by tree; color, alternate-allele fraction); bottom, ground-truth lentiviral clones stratified by size (≥0.5%, 64 clones; <0.5%, 190 clones). Clone recovery accuracy is defined as the fraction of lentiviral clones whose best-matching clade achieves Jaccard overlap >0.5. Filter-2 improves recovery versus Filter-1. The heteroplasmy-based unrefined MitoDrift tree (Manhattan distance) outperforms the binarized ReDeeM tree (weighted Jaccard) given the same mutation filtering strategy.

Table S1: MitoDrift run parameters used across analyses.

Analysis	Dataset	Samples included	MCMC iter	ASDSF thres	Chains	Burn-in
pLARRY tree accuracy benchmark (full tree)	pLARRY	pL1000	150000		50	10000
pLARRY tree accuracy benchmark (subsample trees)	pLARRY	pL1000 (subset seeds 1–10)		0.05	50	2000
WGS benchmark (subsampled trees)	WGS benchmark (Mitchell et al.)	AX001, SX001, KX001–KX004, KX007–KX008 (seeds 1–10)	10000		50	1000
WGS benchmark (full donor trees)	WGS benchmark (Mitchell et al.)	AX001, SX001, KX001–KX004, KX007–KX008	20000		20	2000
MAESTER-CHIP benchmark	MAESTER-CHIP (Miller et al.)	MAESTER-CHIP		0.1	10	10000
Extended ReDeeM cohort mtSDI by lineage (cell-type subsamples)	ReDeeM	Young1, Young2, Young8, Aged1, Aged2 x cell types (seeds 1–20)	35000		20	5000
Multiplexed cohort mtSDI by lineage (hash-refined subsamples)	ReDeeM (multiplexed)	BMMC2, BMMC3, BMMC8, BMMC10, SBM1253, SBM1028, SBM1037 x cell types (seeds 1–10)	5000		20	500
AP-1 in aged HSC and MPP	ReDeeM	Aged1, Aged2 (HSC, MPP)		0.05	50	4000
Young1 longitudinal HSC regulon heritability (refined HSC trees)	ReDeeM	Young1 T1, T2 (refined HSC)		0.1	20	10000
Myeloma joint-timepoint phylogenies	Myeloma multiome	MM1 plasma (all timepoints)		0.1	20	10000
	Myeloma multiome	MM2 plasma (all timepoints)		0.1	10	10000
	Myeloma multiome	MM3 plasma (all timepoints)		0.1	10	10000
In vitro CD34+ differentiation (800-cell subset)	CD34+ differentiation (Lareau et al.)	mtscATAC	350000		10	35000

bioRxiv preprint doi: https://doi.org/10.64898/2026.02.12.705660; this version posted February 18, 2026. The copyright holder for this preprint (which was not certified by peer review) is the author/funder, who has granted bioRxiv a license to display the preprint in perpetuity. It is made available under aCC-BY 4.0 International license.

Table S1. MitoDrift run parameters used across analyses.

Summary of MitoDrift run parameters (datasets, samples included, MCMC iterations, ASDSF threshold, number of chains, and burn-in) used across analyses and figure panels.
